# Type-I Interferons Inhibit Interleukin-10 Signaling and Favor Type 1 Diabetes Development in Nonobese Diabetic Mice

**DOI:** 10.3389/fimmu.2018.01565

**Published:** 2018-07-16

**Authors:** Marcos Iglesias, Anirudh Arun, Maria Chicco, Brandon Lam, C. Conover Talbot, Vera Ivanova, W. P. A. Lee, Gerald Brandacher, Giorgio Raimondi

**Affiliations:** ^1^Vascularized and Composite Allotransplantation Laboratory, Department of Plastic and Reconstructive Surgery, Johns Hopkins School of Medicine, Baltimore, MD, United States; ^2^Institute for Basic Biomedical Sciences, Johns Hopkins School of Medicine, Baltimore, MD, United States

**Keywords:** type-I interferons, interleukin-10 signaling, T lymphocytes, type 1 diabetes, nonobese diabetic mice

## Abstract

Destruction of insulin-producing β-cells by autoreactive T lymphocytes leads to the development of type 1 diabetes. Type-I interferons (TI-IFN) and interleukin-10 (IL-10) have been connected with the pathophysiology of this disease; however, their interplay in the modulation of diabetogenic T cells remains unknown. We have discovered that TI-IFN cause a selective inhibition of IL-10 signaling in effector and regulatory T cells, altering their responses. This correlates with diabetes development in nonobese diabetic mice, where the inhibition is also spatially localized to T cells of pancreatic and mesenteric lymph nodes. IL-10 signaling inhibition is reversible and can be restored *via* blockade of TI-IFN/IFN-R interaction, paralleling with the resulting delay in diabetes onset and reduced severity. Overall, we propose a novel molecular link between TI-IFN and IL-10 signaling that helps better understand the complex dynamics of autoimmune diabetes development and reveals new strategies of intervention.

## Introduction

Type 1 diabetes is a complex autoimmune disease characterized by the progressive destruction of the insulin-producing β-cells in the pancreas by autoreactive T lymphocytes (1). It is caused by a combination of genetic predisposition and environmental factors. In the past decade, viral infections and the composition of the gut microbiota have gained increasing attention as environmental factors that contribute to the initiation of the disease (2–4) but the mechanisms by which these factors contribute to the activity of diabetogenic T cells remains unknown. It is clear that CD4 T cells are of chief importance in this disease. Nonobese diabetic (NOD) mice, a widely used model of human disease that spontaneously develop diabetes, are protected from the disease onset when deficient in CD4 T cells (5, 6), and enriched CD4^+^ cells from diabetic donors are able to transfer the disease when administered into NOD-scid/scid recipients (7). However, the connection between environment and the activity of diabetogenic T cells remains elusive.

Multiple clinical and experimental observations point toward type-I interferons (TI-IFNs), essential cytokines for the clearance of viruses, as the mediators that drive a pre-diabetic or susceptible individual toward type 1 diabetes (8). Relevant examples include high levels of IFN-α detected in the pancreas of diabetic patients (9), absence of autoantibodies able to neutralize IFN-α in the subset of AIRE-deficient (APS1) patients who developed diabetes (10), induction of diabetes in non-autoimmune prone C57BL/6 mice by overexpression of IFN-α in β-cells (11), accumulation of high levels of TI-IFN in NOD mice (12), and delay of disease onset (and decreased incidence) with early blockade of TI-IFN receptor signaling (13). More recently, Ferreira and colleagues reported that an IFN signature in PBMC of genetically predisposed children was detectable before the appearance of islet-specific autoantibodies (14). Despite these observations, the mechanism(s) through which TI-IFN promotes T1D remains poorly understood.

The cytokine interleukin-10 (IL-10) has an essential role in the development of autoimmune pathologies (15). Previous studies suggested that the low expression of this cytokine in the pancreas mediates the occurrence of diabetes (16) and decreased IL-10 levels in serum of newly diagnosed children with type 1 diabetes has been observed (17). Monocytes/macrophages have been historically investigated as the main target of this cytokine (18); however, IL-10 acts also directly on T cells. This has been shown in the context of naïve T cells activation and differentiation (19, 20), in the regulation of effector and memory T cells (Tmem) (21–23), and in the preservation of regulatory T cell (Treg) function (24, 25).

Here, we report a novel effect of TI-IFN that causes a selective inhibition of IL-10 signaling in T cells thereby reducing their capacity to be regulated. This loss of signaling correlates with the development of the disease in NOD mice. This effect is sustained but compartmentalized, manifesting only in T cells of pancreatic lymph nodes (PLN) and mesenteric lymph nodes (MLN) of NOD mice, suggesting a link with the response to the gastric environment in these animals. Importantly, IL-10 signaling in T cells could be partially restored *via* blockade of TI-IFN signaling, supporting earlier observations on the beneficial effects of transient TI-IFN blockade in NOD mice (13). Overall, our results reveal a new molecular mechanism involved in the causative process of type 1 diabetes and suggest novel targets for its prevention and treatment.

## Materials and Methods

### Mice

Nonobese diabetic, wt C57BL/6 (B6), C57BL/6-Foxp3-GFP, IFN-AR1^−/−^, and Rag^−/−^ mice were purchased from Jackson Laboratories, and bred at the Johns Hopkins School of Medicine facility. All animal experiments were conducted in accordance with the National Institutes of Health guide for use and care of laboratory animals, and under a protocol approved by the JHU Animal Care and Use Committee.

### Media, Reagents, and Antibodies

RPMI-1640 and IMDM media were supplemented with 10% v/v heat-inactivated FCS (Atlanta Biologicals, Flowery Branch, GA, USA), 0.1 mM non-essential amino acids, 2 mM l-glutamine, sodium pyruvate, 100 IU/ml penicillin, 100 µg/ml streptomycin, and 50 µM 2-ME (Gibco). Recombinant IFN-β and IFN-α were purchased from PBL Assay Science. Blocking anti-IFN-AR1 mAb was from Leinco Technologies (St. Louis, MO, USA). Recombinant IL-10 and IL-6 were from PeproTech (Rocky Hill, NJ, USA). Jak inhibitors Tofacitinib and Ruxolitinib were purchased from LC Laboratories (Woburn, MA, USA).

### T Cell (Subsets) Isolation

Spleen and lymph nodes were harvested and total/CD4 T cells were isolated *via* magnetic-bead negative selection. Briefly, cells were incubated with anti-mouse Ter119 (TER-119), Gr1 (RB6-85C), CD11b (M1/70), B220 (RA3-6B2), CD16/32 (2.462), I-A/I-E (M5/114.15.2) [also anti-CD8 (53-6.7) for CD4 T cell purification] (all from BD Biosciences) followed by incubation with magnetic beads conjugated with anti-rat IgG (ThermoFisher) at a 1:1 (cell:bead) ratio. The resulting total/CD4 T cells were >90% pure. Where indicated, Treg (CD4^+^CD25^+^) were isolated from CD4 T cells following the protocol described in the EasySep PE-selection kit (STEMCELL technologies).

### CD4 Tmem Generation

In some experiments, Tmem were generated *via* a modification of the published “parking method” (26). Briefly, 20 × 10^7^ T cells from B6 mice were activated with anti-CD3 (0.5 µg/ml; BD Pharmingen) in the presence of syngeneic LPS-matured bone marrow-derived DCs (1:20 ratio DC:T cell) as previously described (27). Three days later, activated T cells together with 10^7^ T cell-depleted splenocytes [obtained *via* removal of CD3^+^ cells from single cell suspensions using the protocol described in Section “T Cell (Subsets) Isolation”] were infused intravenously into Rag^−/−^ mice. Four weeks later, Tmem were isolated and used for the indicated experiments.

### Cell Stimulation and Preparation for Phospho-Flow Analysis

For assessment of proteins phosphorylation *via* flow cytometry (Phospho-flow), a modification of the protocol published by the Nolan group (28) was utilized. Briefly, 10–15 × 10^6^ purified T cells were cultured in IMDM complete media with/without IFN-β (1–25 ng/ml) for indicated periods and then rested in cytokine-free media for six additional hours. 2 × 10^6^ fresh/cultured cells were stimulated for 20 min with IL-10 (40 ng/ml) or IL-6 (40 ng/ml), or 30 min with IFN-β/α (5 ng/ml). Then, cells were fixed for 50 min by adding 2.4 ml of a solution containing 4% paraformaldehyde and 1.4% methanol in PBS. After fixation, 600 µl of 1× wash buffer (contained in the Transcription Factor Phospho Buffer Set kit, BD Biosciences) were added to the previous mixture, mixed, and spun down. Finally, cells were suspended with 500 µl Perm Buffer III (BD Biosciences) while vortexing, and stored at −20°C until use.

### Flow Cytometry and Cell Sorting

In Phospho-flow experiments, Perm Buffer III was removed and cells stained with fluorchrome-labeled antibodies against CD4 (RM4-5), CD44 (IM7), Foxp3 (FJK-1) (Thermo Fisher eBioscience), and Stat3 (pY705) (4/P_STAT3; BD Phospho-flow). For IL-10R staining, the Human IL-10 biotinylated Fluorokine kit (R&D Systems) was used. Detection of suppressor of cytokine signaling protein (SOCS)1 and SOCS3 mRNA levels *via* flow cytometry was performed employing the PrimeFlow RNA assay kit (Thermo Fisher) following the manufacturer guidelines. Data were acquired using an LSR-II flow cytometer (BD Biosystems) and analyzed with FlowJo X version software (FLOWJO, LLC, Ashland, OR, USA). For WB and qPCR experiments using T cell subpopulations, fresh or cultured T cells were stained with CD4 (RM4-5), CD25 (PC61) (not in the case of cells from Foxp3-GFP mice), and CD44 (IM7) antibodies and the specific subsets of Tmem (CD4^+^Foxp3^−^CD44^+^) and Treg (CD4^+^Foxp3/GFP^+^ or CD4^+^CD25^+^) were sorted to 99% purity using a FACS Aria II cell sorter.

### Western Blot

Either total CD4 T cells or Tmem and Treg subpopulations after specific culture conditions were lysed with RIPA buffer containing proteases and phosphatases inhibitors. MG-132 (Millipore-Sigma) was added for the last 1.5 h of culture in the experiments where SOCS protein levels were studied. Cell lysates were run in 10–12% acrylamide gels and proteins transferred to a PVDF membrane. Protein expression levels were detected with the following antibodies: phospho-STAT3 (Y705) (D3A7), SOCS1 (A156), SOCS3 (L210) (all from Cell Signaling Technology), and β-actin (I-19; Santa Cruz biotechnology). Protein expression was detected using fluorescent-labeled secondary antibodies (LI-COR). Data were acquired using an Odyssey CLx (LI-COR) imaging system and analyzed with ImageJ software to normalize values to β-actin levels.

### Quantitative Real-Time PCR

CD4 T cells were lysed in TRIzol reagent (Thermo), and RNA was extracted using chloroform (Fisher Scientific) and the RNeasy MiniElute Cleanup Kit (Qiagen). The mRNA was reverse transcribed using the SuperScript IV First Strand Synthesis System and protocol (Thermo Scientific). Real-time RT-PCR was performed on a QuantiStudio 12K Flex Real-Time PCR system (Thermo Scientific) calibrated for SYBR Green detection. The primers and conditions employed are listed in Table S1 in Supplementary Material.

### Statistical Analysis

Differences in flow cytometry quantification of P-STAT3-mean fluorescence intensity (MFI) were analyzed using either two-sample Mann–Whitney *U* test or two-tailed paired Student’s *t*-test. To minimize the impact of fluctuations in fluorescence among experiments, the coefficient index (MFI P-STAT3 in stimulated cells/MFI P-STAT3 in unstimulated cells) was calculated and averaged for each experiment and then used for statistical analysis. P-STAT3 expression comparisons between T cells of NOD and B6 mice were analyzed using two-way ANOVA. Two-tailed unpaired Student’s *t*-test was applied to test gene expression differences from PCR experiments. All analyses were performed with Prism Software version 5.0 (GraphPad, La Jolla, CA, USA).

## Results

### Localized Defective IL-10 Signaling in Memory and Regulatory CD4 T Cells in TI-IFN Enriched Tissues

**Figure 1:**
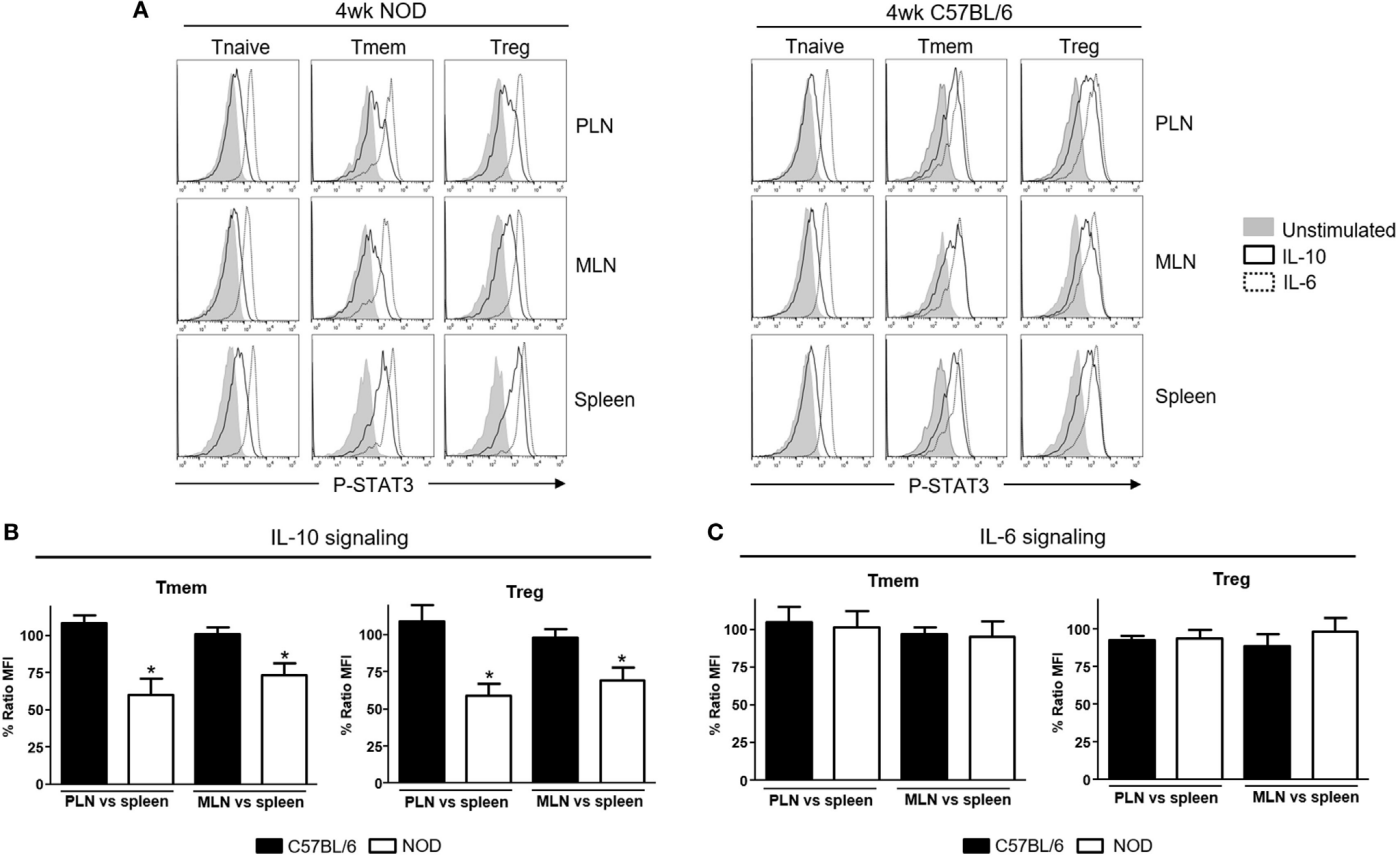
Defective interleukin-10 (IL-10) signaling in memory T cells (Tmem) and regulatory T cells (Tregs) in pancreatic and mesenteric lymph nodes of nonobese diabetic (NOD) mice. Cells of pancreatic lymph nodes (PLN), mesenteric lymph nodes (MLN), and spleens of 4-week-old NOD or C57BL/6 mice were either left untreated or stimulated with IL-10 (40 ng/ml) or IL-6 (40 ng/ml) for 20 min. Phosphorylated-STAT3 (P-STAT3) levels induced in CD4 T cell subpopulations (Tnaive: CD4^+^CD44^low^Foxp3^−^, Tmem: CD4^+^CD44^hi^Foxp3^−^, and Treg: CD4^+^Foxp3^+^) were measured by Phospho-flow. **(A)** Representative histograms of P-STAT3 levels in the indicated CD4 T cell subpopulations after IL-10 and IL-6 stimulation. **(B,C)** Cumulative representation of the percentage of P-STAT3 signal induction in PLN/MLN Tmem and Treg after IL-10 **(B)** or IL-6 **(C)** stimulation compared to level induced in the splenic populations of the indicated mouse strain. This ratio of mean fluorescence intensities (MFIs) was calculated by comparing the coefficient index of P-STAT3 between two different tissues, considering the levels in spleen as 100% of expression. Data shown in **(B,C)** are the average from *n* = 4 mice per strain and are expressed as % of ratio MFI ± SEM, *p < 0.05, Mann–Whitney *U* test.

Li et al. reported unexpectedly high levels of IFNα production in the PLN of NOD mice starting in the second week of life that correlated with the presence of CD4 T cells with a transcriptional signature abundant in INF-induced genes (13). We examined *via* qPCR the expression of different interferons transcripts (Figure S1 in Supplementary Material) and found that IFN-α (IFNα4 and IFNα9) but also IFN-β1 mRNA levels were upregulated in PLN of 4-week-old NOD mice when compared to the levels found in the spleen. This upregulation was maintained at least until the 12^th^ week of life. Based on the suspected involvement of IL-10 in disease development, we tested if these TI-IFN-exposed T cells would show any alteration in their response to IL-10. To evaluate signal integrity, we quantified the accumulation of the phosphorylated (active) form of the transcription factor STAT3 (P-STAT3, a key molecule in the IL-10 signaling pathway) in response to *ex vivo* stimulation with IL-10. The response to the pro-inflammatory cytokine IL-6 (that also induces phosphorylation of STAT3) was measured to distinguish between cytokine-specific vs nonspecific effects of TI-IFN exposure. We compared multiple CD4 T cell subsets: naïve (CD4^+^CD44^low^Foxp3^−^), memory (CD4^+^Foxp3^−^CD44^hi^), and regulatory (CD4^+^Foxp3^+^) T cells residing in PLN, MLN, axillary lymph nodes (ALN), and spleen (each separately) in 4-week-old NOD mice—the time of highest accumulation of IFNα and IFNβ [(13) and our data]. Independent repeats of these measurements indicated a statistically significant reduction in IL-10 signaling in both Tmem and Treg from PLN and also from MLN compared to the response of the same T cell subsets in the spleen (Figures 1A,B) and ALN (not shown). The reduced response in MLN paralleled the observed accumulation of TI-IFNs in these lymphoid tissues (Figure S1 in Supplementary Material). In 4-week-old non-diabetes prone B6 mice, which do not accumulate TI-IFN in pancreatic and mesenteric lymph nodes (Figure S1 in Supplementary Material), Tmem and Treg preserved their ability to fully respond to IL-10 in all lymphoid tissues (Figures 1A,B). Importantly, this decrement in STAT3 phosphorylation was specific to IL-10 signaling, as the response to IL-6 was unaltered in the T cells of NOD (and B6) mice from all the lymphoid tissues tested (Figure 1C). Together, these results suggested that in NOD mice there is a selective reprogramming of the signaling for IL-10, actuated specifically in lymphoid tissues shown to accumulate TI-IFN (13).

### The Impact of TI-IFN on IL-10 Signaling Is Not a Genetic Characteristic of NOD T Cells

**Figure 2:**
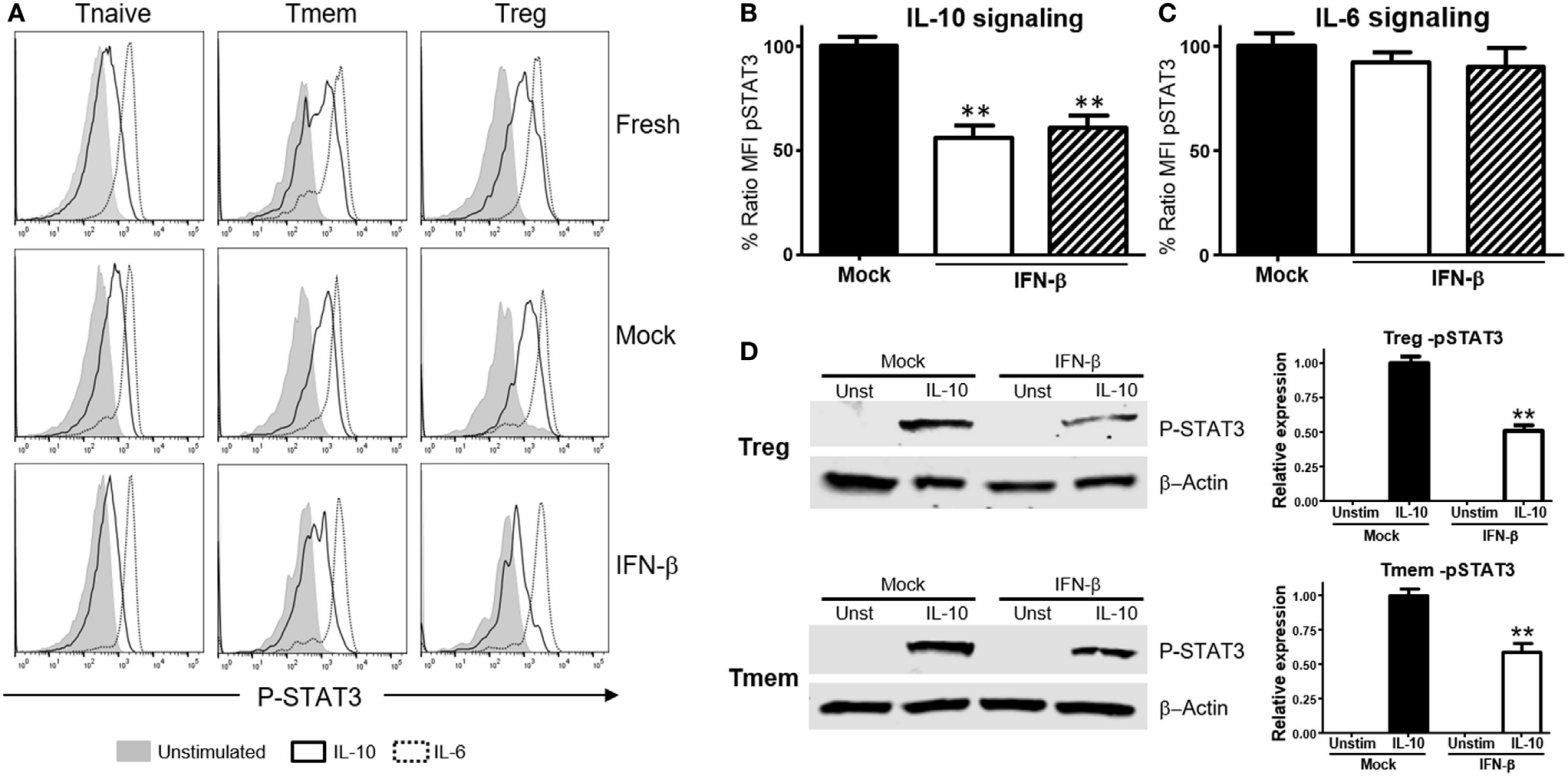
Exposure to IFN-β induces a selective inhibition of interleukin-10 (IL-10) signaling in CD4 memory T cells (Tmem) and regulatory T cell (Treg) irrespective of the strain of origin. **(A–C)** Purified T cells from C57BL/6 mice were freshly stimulated or cultured for 48 h in complete media with or without IFN-β (5 ng/ml), and rested in cytokine-free media for six additional hours. Cells were then either left untreated or stimulated with IL-10 (40 ng/ml) or IL-6 (40 ng/ml) for 20 min, and the levels of P-STAT3 in CD4 T cell subpopulations were measured by Phospho-flow as in Figure 1. **(A)** Representative histograms of the mean fluorescence intensity (MFI) levels of P-STAT3 in the different CD4 T cell subpopulations of fresh, mock, and IFN-β exposed T cells after IL-10 and IL-6 stimulation. **(B,C)** Graph bars that compare the percentage of P-STAT3 MFI ratio between IFN-β exposed and not exposed (mock) cultured Tmem and Tregs after IL-10 **(B)** or IL-6 **(C)** stimulation. Ratio MFI was calculated comparing the coefficient index of P-STAT3 after stimulation between the two different culture conditions, considering levels in mock cells as 100% of expression. Data from *n* = 6 individual experiments are shown and expressed as % of ratio MFI ± SEM, **p < 0.01, Mann–Whitney *U* test. **(D)** T cells from C57BL/6-Foxp3-GFP mice were cultured for 48 h in complete media with or without IFN-β (5 ng/ml), and then Tmem (CD4^+^Foxp3-GFP^−^CD44^+^) and Treg (CD4^+^Foxp3-GFP^+^) subpopulations were flow sorted. After a 6 h resting phase, cells were left untreated or stimulated with IL-10 (40 ng/ml) for 20 min, lysed and the levels of pSTAT3 compared to β-actin were measured by Western Blot. Data from *n* = 3 individual experiments are shown and expressed as a relative expression with the control ± SEM, **p < 0.01, Student’s *t*-test.

We then tested whether this effect was unique to T cells of NOD background, or bystander exposure of any T cells to unusual levels of TI-IFN could affect their ability to be controlled by IL-10. Bulk T cells from wt B6 mice were exposed to IFN-β or IFN-α for 48 h, and then the levels of P-STAT3 induced by stimulation with IL-10 or IL-6 were quantified *via* Phospho-flow. Exposure to IFN-β induced a statistically significant reduction of STAT3 phosphorylation after IL-10 stimulation in Tmem and Tregs when compared to the response in fresh or mock-treated cells (cultured without IFN-β, to exclude any impact from the culturing conditions) (Figures 2A,B). These results were confirmed *via* western blot measurement of phospho-STAT3 levels (Figure 2D). Reduction of IL-10 signaling was IFN-β dose-dependent, reaching maximum inhibition at 5 ng/ml of IFN-β (Figure S2A in Supplementary Material). Exposure to IFN-α induced a similar inhibition of IL-10 signaling in a dose-dependent manner, though it required a higher concentration for maximal inhibition than IFN-β (Figure S2B in Supplementary Material). As observed in T cells from NOD mice, the levels of P-STAT3 in response to IL-6 stimulation remained unaltered under all conditions (Figures 2A,C), confirming that this effect was not a generalized saturation of the Jak/STAT signaling pathway.

### IFN-β-Mediated Inhibition of IL-10 Signaling Alters Induction of IL-10 Responsive Genes

**Figure 3:**
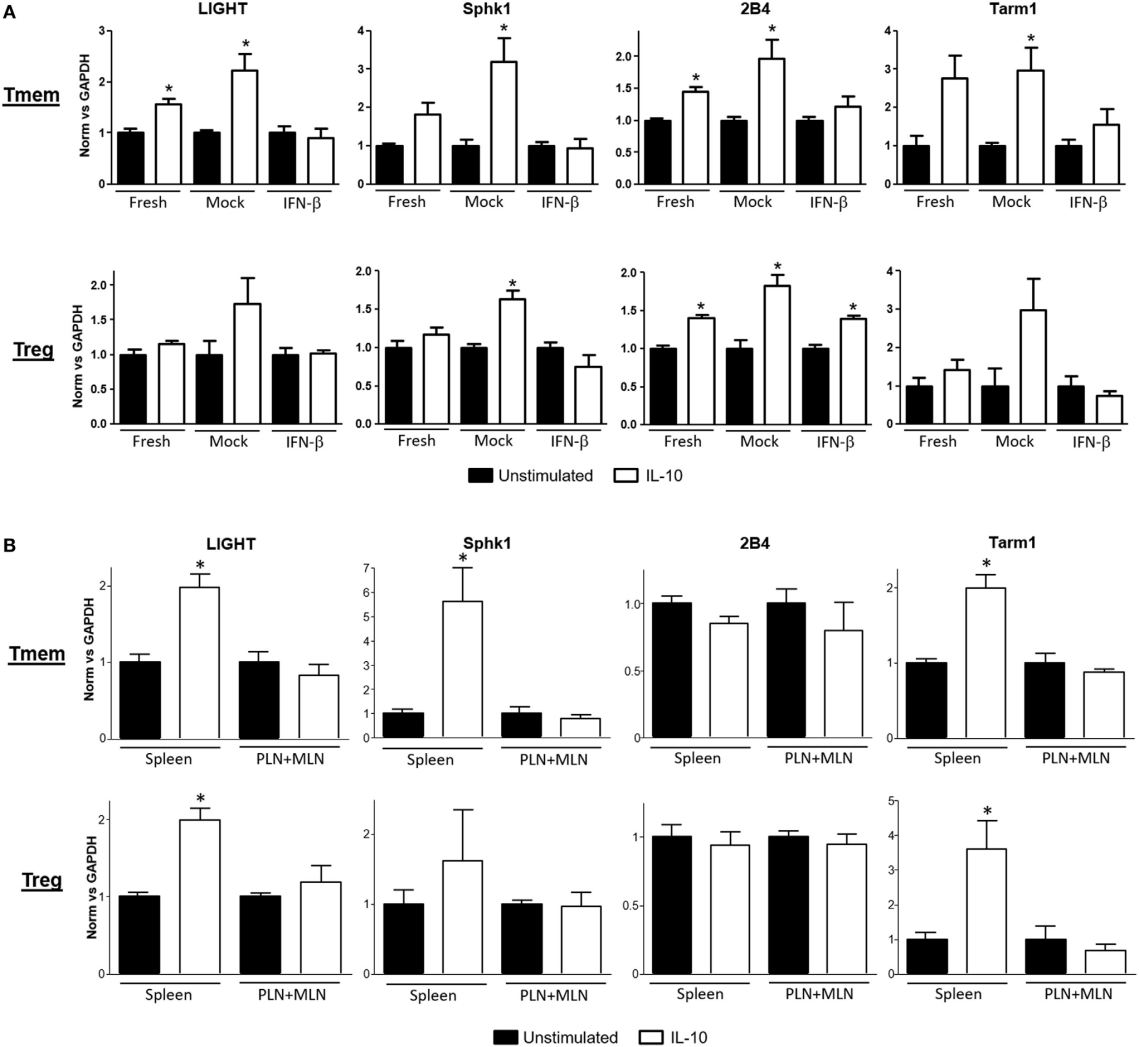
Expression of interleukin-10 (IL-10) regulated genes in T cells is abrogated after exposure to IFN-β. **(A)** Freshly purified CD4 memory T cells (Tmem) (obtained using the “parking method”; see Materials and Methods) and CD4^+^CD25^+^ regulatory T cells (Tregs) from C57BL/6 mice were left untreated or cultured 48 h without (mock) or with IFN-β (5 ng/ml). After an additional 4 h of resting (without stimuli), cells were either left untreated or stimulated for 4 h with IL-10 (40 ng/ml). **(B)** Freshly purified Tmem and Treg (CD4^+^CD25^+^) from spleen and pooled pancreatic lymph nodes (PLN) + mesentheric lymph nodes (MLN) were either left untreated or stimulated for 4 h with IL-10 (40 ng/ml). **(A,B)** Cells were then lysed and mRNA levels of *LIGHT, Sphk1, 2B4*, and *Tarm1* genes were measured by qPCR. ΔΔCt method was used to calculate their relative expression and then normalized to GAPDH. The graph bars show the fold change between unstimulated and stimulated conditions in the different groups ± SEM. *p < 0.05 in unpaired Student’s *t*-test was considered statistically significant. Data show the average of *n* = 3 independent experiments **(A)** or *n* = 4 animals per group **(B)**.

A deeper evaluation of the functional impact that inhibition of IL-10 signaling by TI-IFN has on the modulation of T cells can be done *via* assessment of its transcriptional impact. However, the transcriptional impact of IL-10 on T cells is unknown. We therefore harnessed the vast knowledge about IL-10 signaling in antigen-presenting cells. Taking advantage of data from the most recent publicly available RNAseq analysis of mouse macrophages exposed to IL-10 (29), we selected a pool of 29 genes highly upregulated (>6σ) (Figure S3A in Supplementary Material) as initial lead for genes that could be also induced in T cells. Ten of these upregulated genes had known functions in T cells (Figure S3B in Supplementary Material). We then tested the expression of these genes as a screening panel for further investigation of Treg and Tmem cells responses to IL-10 with or without TI-IFN pre-exposure. To obtain a sufficient and homogeneous number of Tmem, we implemented the previously published “parking method” (see Materials and Methods) where *in vitro*-activated T cells are “parked” in congenic Rag^−/−^ mice to generate Tmem (26). Tregs were freshly isolated from unmanipulated animals. Gene transcription analysis showed four genes—*LIGHT* (Tnfsf14), *Sphk1, Tarm1*, and *2B4*—to be significantly upregulated in Tmem by *in vitro* treatment with IL-10 (Figure 3A), and two genes, *Sphk1* and *2B4*, were upregulated in Treg. We then analyzed if the induction of these genes was affected by pre-exposure of these cells *in vitro* to IFN-β. In Tmem, the increased expression of *LIGHT, Sphk1, Tarm1*, and *2B4* was completely abrogated. mRNA levels of *Sphk1, LIGHT*, and *Tarm1* also showed an important decrease in IFN-β exposed Treg, while the expression of *2B4* was not affected. Supported by these results, we determined whether the *in vivo* exposure of NOD T cells to normal (spleen) or high levels of TI-IFN (PLN and MLN) affected the induction of these genes by IL-10. Tmem and Tregs were flow sorted from spleen and from pooled PLN + MLN and then stimulated *ex vivo* with IL-10 (Figure 3B). Tmem cells from the spleen showed a significant upregulation of *LIGHT, Sphk1, and Tarm1*, while all four genes could not be induced by IL-10 in cells from PLN + MLN. In NOD Treg, IL-10 induced a statistical increase in *LIGHT and Tarm1* in cells from the spleen while, again, none of them was upregulated in cells from PLN + MLN. These results indicate that the selective inhibition of STAT3 phosphorylation induced by INF-β pre-exposure alters significantly the impact of IL-10-induced transcription in Tmem and Tregs both *in vitro* and *in vivo*.

### Inhibition of IL-10 Signaling Requires Prolonged Exposure to IFN-β, but It Is Reversible

**Figure 4:**
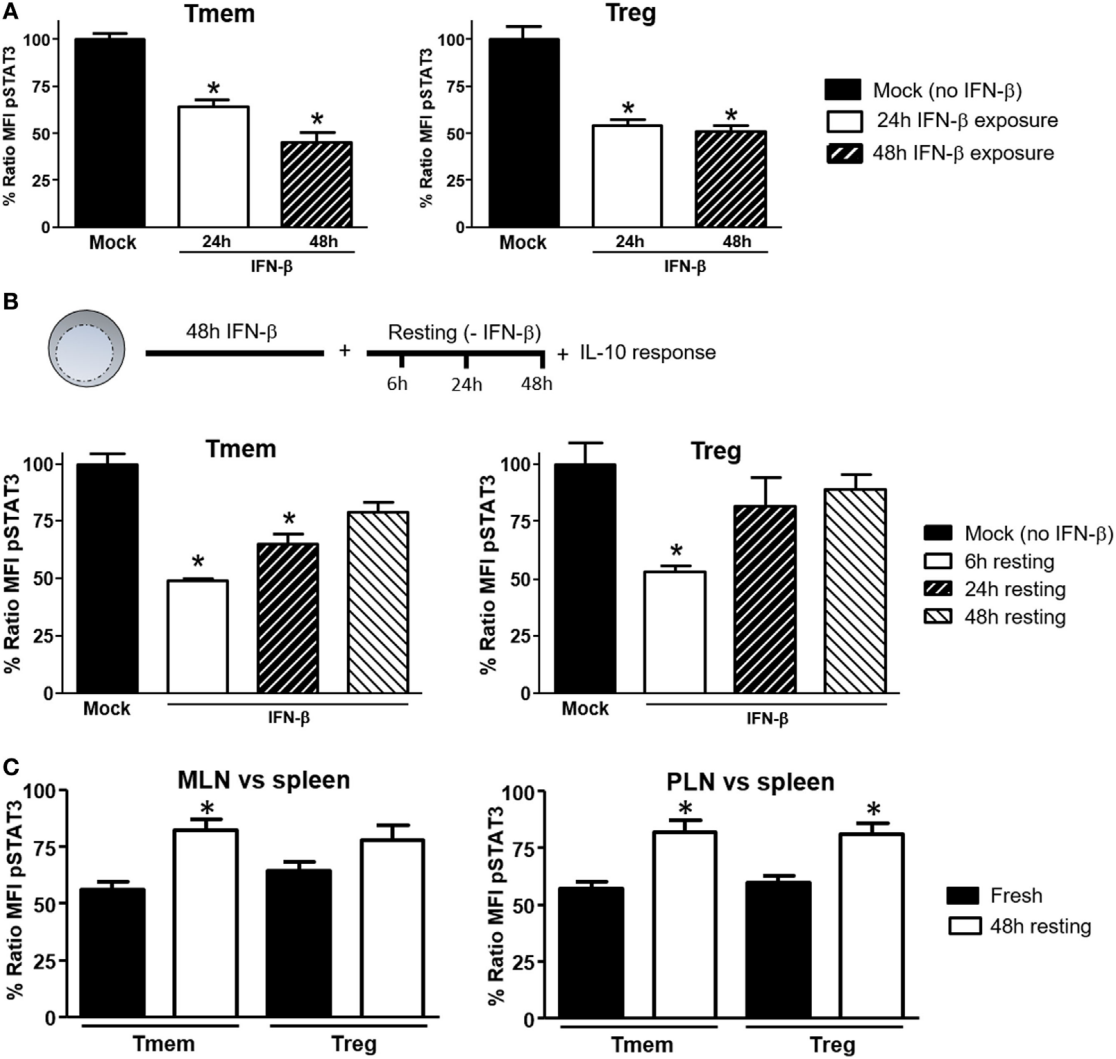
Induction of interleukin-10 (IL-10) signaling inhibition in T cells requires prolonged exposure to IFN-β but is reversible. **(A)** Purified T cells from C57BL/6 mice were cultured for 24 or 48 h with or without IFN-β (5 ng/ml) and rested in cytokine-free media for six additional hours. Cells were then left untreated or stimulated with IL-10 (40 ng/ml) and the inhibition of P-STAT3 induction by IL-10 assessed as indicated in Figure 1. **(B)** Purified T cells from C57BL/6 mice were cultured for 48 h with or without IFN-β (5 ng/ml) and then rested in cytokine-free media for the indicated time (6, 24, and 48 h). The levels of P-STAT3 in response to IL-10 (40 ng/ml) were then assessed as previously indicated. The ratio mean fluorescence intensity (MFI) **(A,B)** was calculated comparing the coefficient index of P-STAT3 after IL-10 stimulation between IFN-β exposed and mock, considering levels in mock cells as 100% of expression. Data of *n* = 4 individual experiments are shown and expressed as % of ratio MFI ± SEM, *p < 0.05, Mann–Whitney *U* test. **(C)**. Cells from pancreatic lymph nodes (PLN), mesentheric lymph nodes (MLN), and spleens of 4- to 6-week-old nonobese diabetic (NOD) mice were either freshly stimulated or cultured in cytokine-free media for 48 h and then stimulated with IL-10 (40 ng/ml) for 20 min. The levels of P-STAT3 in response to IL-10 (40 ng/ml) were then assessed as previously indicated. Cumulative results are shown in the graph where bars represent the normalized P-STAT3 MFI ratio in memory T cells (Tmem) or regulatory T cell (Treg) after IL-10 stimulation between the spleen and PLN or MLN in fresh vs rested conditions. Ratio MFI was calculated by comparing the coefficient index of P-STAT3 between the two different organs, considering the levels in spleen as 100% of expression. Data of *n* = 4 mice per group are shown and expressed as % of ratio MFI ± SEM, *p < 0.05, Mann–Whitney *U* test.

To explore what length of exposure to IFN-β is required to impact IL-10 signaling in T cells, wt B6 bulk T cells were exposed to IFN-β for different lengths of time and the response to IL-10 and IL-6 (not shown) in different subsets assessed by Phospho-flow. In Tmem cells, a 24-h exposure significantly reduced the levels of IL-10-induced P-STAT3, but a 48-h exposure was necessary to achieve maximal inhibition (Figure 4A). In Tregs, a 24-h exposure was sufficient to achieve the maximal inhibition of IL-10-induced P-STAT3 signaling (Figure 4A).

We also tested the reversibility of this inhibition. To address this question, after 48 h of exposure to IFN-β, T cells were washed, rested in cytokine-free media for 24 or 48 h and the P-STAT3 response to IL-10 or IL-6 (not shown) was then measured. Within 24 h of removing IFN-β, Treg recovered their normal P-STAT3 response to IL-10 (Figure 4B). The recovery of Tmem was slower, showing only partial restoration of the IL-10 signaling even at 48 h after removing the IFN-β (Figure 4B). We then determined if the reversibility of inhibition applied also to NOD T cells from PLN and MLN, where they are exposed to high concentrations of a cocktail of TI-IFNs (and possibly for a long period of time). Total cells from spleen, PLN, and MLN of NOD mice were freshly isolated, rested in cytokine-free media for 48 h, and the P-STAT3 response to IL-10 was then measured. Results showed a significant recovery in both Treg and Tmem cells from PLN and MLN rested in IFN-β free media (Figure 4C). These results indicate that the bystander effect of IFN-β requires a prolonged exposure to instigate inhibition of IL-10 signaling and, with some kinetic differences between Treg and Tmem, a normal P-STAT3 response to IL-10 in T cells can be restored following removal of IFN-β.

### IFN-β Signals Through the Jak/STAT Pathway to Inhibit IL-10 Signaling in T Cells

**Figure 5:**
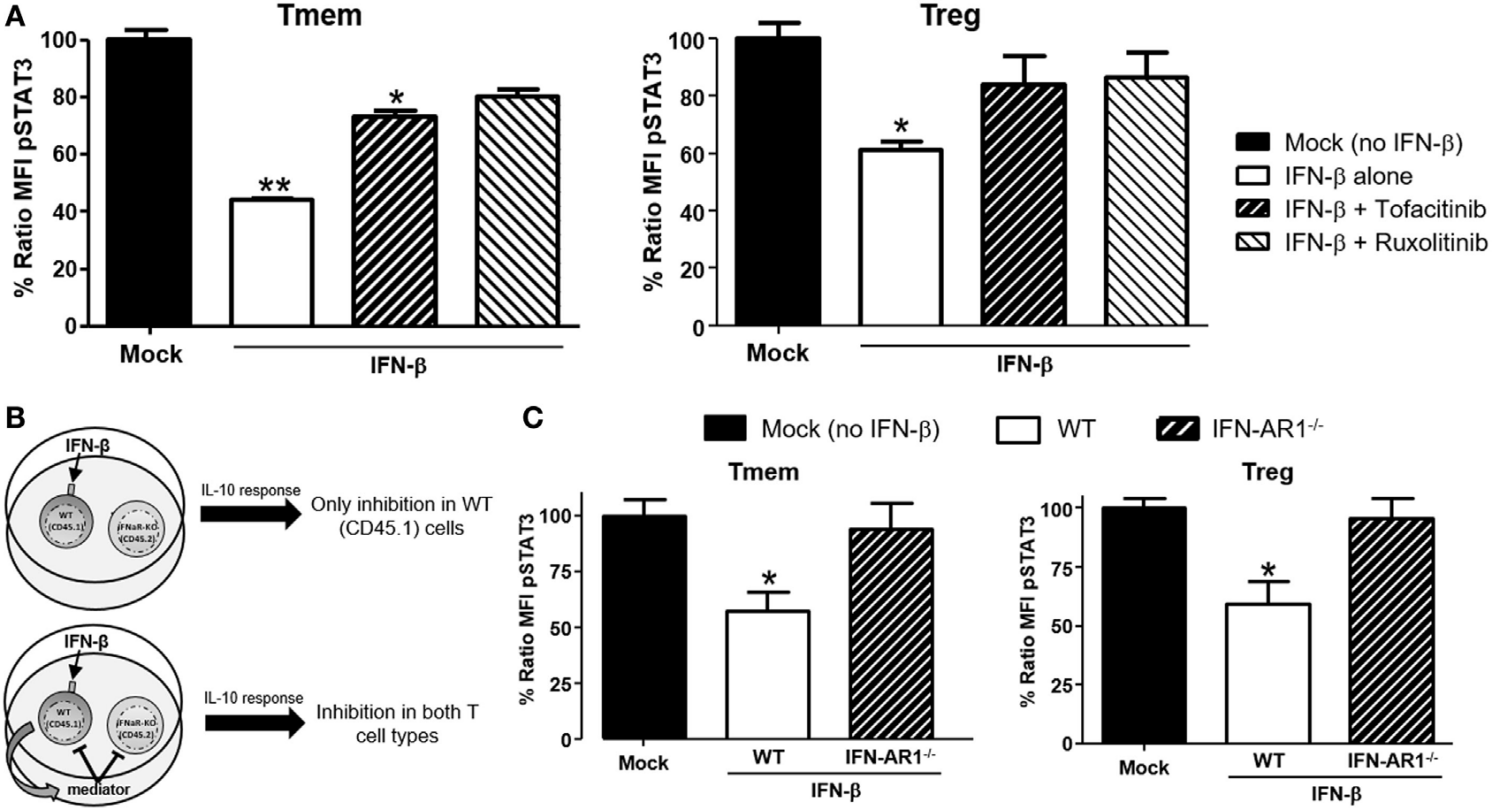
IFN-β signals through JAK–STAT pathway to directly inhibit interleukin-10 (IL-10) signaling in T cells. **(A)** Impact of JAK–STAT inhibition on the ability of IFN-β to modulate IL-10 signaling. T cells from C57BL/6 mice were exposed to Tofacitinib (25 µM) or Ruxolitinib (5 µM) for 2 h before addition of IFN-β and then cultured for 48 h (followed by a 6–8 h resting phase in cytokine-free media). Their ability to respond to IL-10 (40 ng/ml) was then measured by means of P-STAT3 levels assessed *via* Phospho-flow. **(B)** Schematic representation of the experimental approach adopted to assess the direct or indirect impact of IFN-β. **(C)** Purified T cells from congenic C57BL/6 (CD45.1) and IFN-AR1^−/−^ (CD45.2) mice were mixed at a 1:1 ratio. This mix was cultured for 48 h with or without IFN-β (5 ng/ml) and then rested in cytokine-free media for additional 6 h. The response to IL-10 of each subpopulation was then assessed *via* Phospho-flow. Data of *n* = 3 individual experiments are shown and expressed as % of ratio mean fluorescence intensity (MFI) ± SEM, *p < 0.05, **p ≤ 0.01, paired Student’s *t*-test.

Type-I interferons signal through multiple pathways (30), with the activation of the Jak/STAT route considered the most relevant to their antiviral effects. To identify the signaling pathway responsible for the actuation of IFN-β-induced perturbations of IL-10 signaling, cells were pre-treated with two small molecule Jak-specific inhibitors, Tofacitinib and Ruxolitinib, prior to and during IFN-β stimulation. Tofacitinib inhibits Jak3 and Jak1, while Ruxolitinib blocks Jak2 and Jak1. After preconditioning bulk T cells with Tofacitinib (25 µM) or Ruxolitinib (5 µM) for 2 h, IFN-β was added to the cultures for 48 h. Following incubation and washing, an additional 6–8 h resting phase allowed the cells to recover their signaling after removal of the Jak inhibitor (Figure S4 in Supplementary Material). The impact on IL-10 or IL-6 signaling was then quantified *via* phospho-flow. In Tmem, Tofacitinib treatment resulted in a statistically significant preservation of IL-10 signaling and Ruxolitinib had an even stronger effect (Figure 5A). In Treg, both inhibitors restored IL-10 signaling to the same extent (Figure 5A), though the cells had to be rested for 8 h (instead of 6 h as in the case of Tmem), as their recovery of cytokines signaling after Jak inhibition was slower than in Tmem. Collectively, these results suggest that Jak1, and possibly Jak2, are essential mediators for IFN-β-mediated alterations of IL-10-induced P-STAT3 signaling in T cells.

Our data indicate that TI-IFN causes inhibition of IL-10 signaling through a process that requires 24/48 h of exposure. This suggests that multiple intracellular molecular modifications are needed to achieve this phenotype and the process could require the synthesis and activity of additional extracellular mediators. To test this hypothesis, we employed a co-culture system with T cells deficient for the receptor of TI-IFN (IFN-AR1^−/−^; unable to respond to IFN-α or -β). These cells have a response to IL-10 comparable to that displayed by wild type cells (Figure S5 in Supplementary Material). We then exposed B6 wild-type congenic T cells (CD45.1^+^ from B6/SJL mice) mixed at 1:1 ratio with B6-IFN-AR1^−/−^ T cells (expressing the CD45.2 isoform) to IFN-β. If the TI-IFN-induced inhibition of IL-10 signaling requires the synthesis and secretion of an intermediate factor, this molecule would also affect the response of IFN-AR1^−/−^ T cells in our co-culture system (Figure 5B). Phospho-flow analysis indicated that while wild-type Tmem and Tregs showed a reduction of IL-10 signaling after IFN-β exposure, the co-cultured IFN-AR1^−/−^ cells remained completely unaffected (Figure 5C). These results demonstrate that IFN-β acts directly on T cells to condition IL-10 signaling.

### Alteration of Surface IL-10 Receptor Expression or Induction of SOCS Molecules Are Not Responsible For Inhibition of IL-10 Signaling

**Figure 6:**
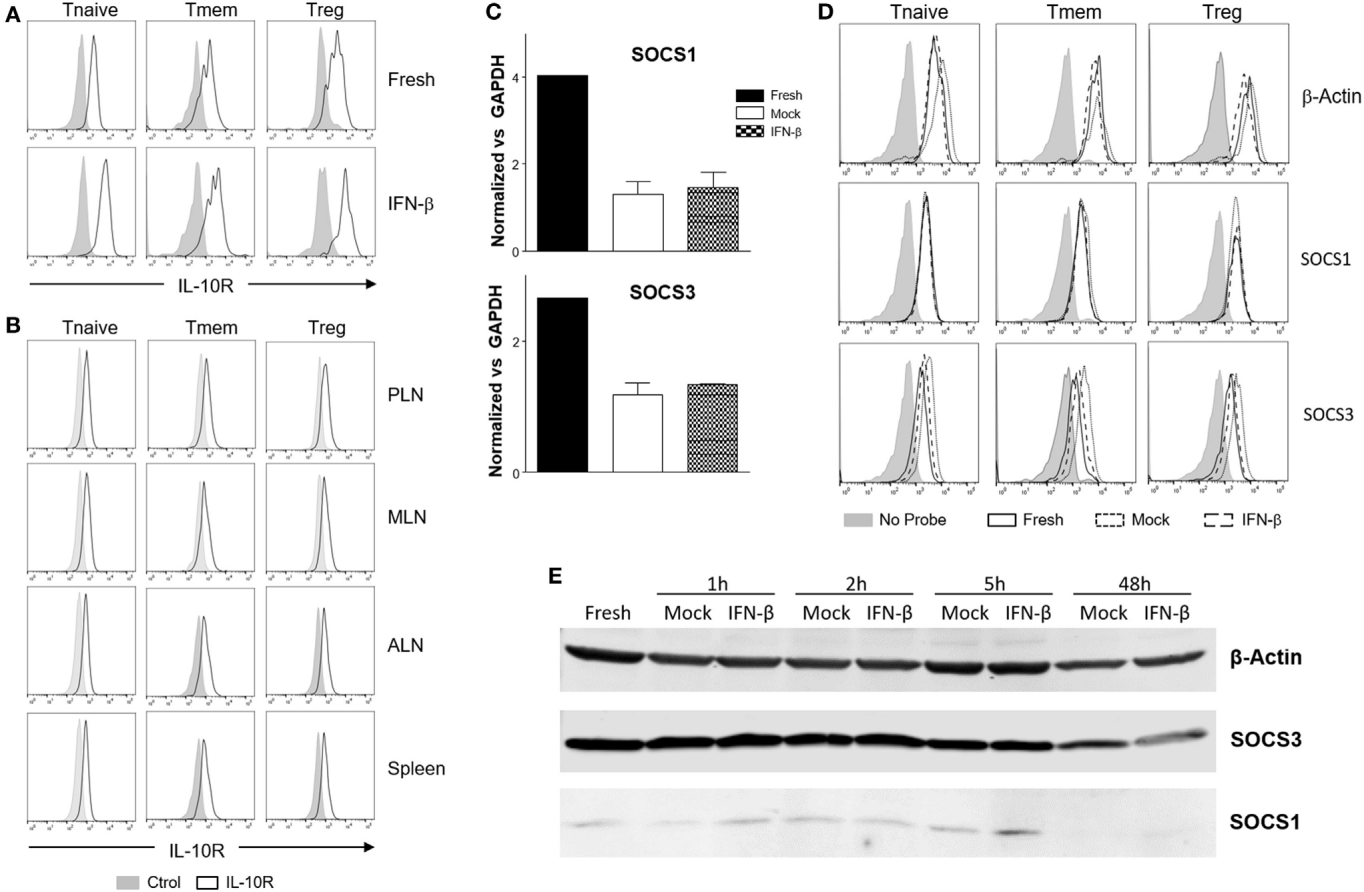
Alteration of surface interleukin-10 (IL-10)R expression or induction of suppressor of cytokine signaling protein (SOCS) molecules are not responsible for the inhibition of IL-10 signaling induced by IFN-β. **(A,B)** T cells from C57BL/6 mice (freshly isolated or post *in vitro* exposure to IFN-β) **(A)** or total cells from pancreatic lymph nodes (PLN), mesentheric lymph nodes (MLN), axillary lymph nodes (ALN), and spleen of 4-week-old nonobese diabetic (NOD) mice **(B)** were stained for their expression of IL-10R in the indicated CD4 T cell subpopulation by flow cytometry. **(C–E)** Purified CD4 memory T cells (Tmem) (obtained *via* the “parking method”) and regulatory T cell (Treg) **(C)**, or bulk T cells from C57BL/6 mice **(D,E)** were left untreated or cultured for the indicated time lengths with or without IFN-β (5 ng/ml) and the mRNA **(C,D)** or protein levels **(E)** of SOCS1 and SOCS3 assessed by qPCR **(C)**, Prime FlowRNA **(D)**, and Western blot **(E)**. In **(C)**, graph bars show qPCR results, calculated using ΔΔCt method and normalized with GAPDH expression. Data of *n* = 3 individual experiments are shown and expressed as a fold change ± SEM. In **(D)**, histograms show mRNA levels of SOCS1 and SOCS3 in CD4 T cell subpopulations in the indicated conditions; levels of β-actin are also shown as a control. Representative results of *n* = 2 experiments are shown. In **(E)**, MG-132 was added for the last 1.5 h of the culture and results were compared with β-actin expression.

Downregulation of IL-10R surface expression would be a plausible mechanism to account for the inhibition of IL-10 signaling following exposure to TI-IFN. However, flow cytometric analysis of IL-10R surface expression did not support this hypothesis. IFN-β exposed T cells (both Tmem and Treg), expressed levels of the receptor comparable to that of non-exposed cells (Figure 6A). In line with this, the comparison of IL-10R expression between NOD T cells isolated from the PLN, MLN, ALN, and spleen showed no differences in the MFI (Figure 6B). This result suggested the involvement of an IL-10R-specific regulator acting between the receptor and STAT3 (as STAT3 remains available for the IL-6 receptor to be phosphorylated). SOCSs act as cytokine-inducible negative regulators of cytokine signaling. We tested if the transcriptional levels of SOCS1 (the only regulator reported in the literature to be associated with inhibition of IL-10 signaling in a lymphoma cell line) (31) and SOCS3 (as control; it does not inhibit IL-10R) were increased by IFN-β treatment. Despite a reduction in the SOCS1 and SOCS3 RNA levels in cultured T cells compared to fresh cells, the transcription levels of SOCS1 and SOCS3 were not increased by IFN-β treatment (Figure 6C). These results were confirmed in Tmem and Treg subpopulations *via* Prime FlowRNA (Affymetrix)—technology that allows detection *via* flow cytometry of RNA and protein expression simultaneously at single cell level—clearly showing that the levels of SOCS1 and SOCS3 RNA were not upregulated in Tmem and Tregs exposed to IFN-β (Figure 6D). Finally, we assessed the level of SOCS1 and SOCS3 protein *via* western blot at both early time point after addition of IFN-β as well as at the investigated 48 h (Figure 6E). Results indicate that despite IFN-β induces a modest increase in SOCS1 levels in the first few hours of stimulation, such increase is not sustained at 48 h and excludes the involvement of SOCS1 in IL-10 signaling inhibition.

### The Localized Defective Response to IL-10 in Tmem and Tregs of NOD Mice Is Maintained With Age

**Figure 7:**
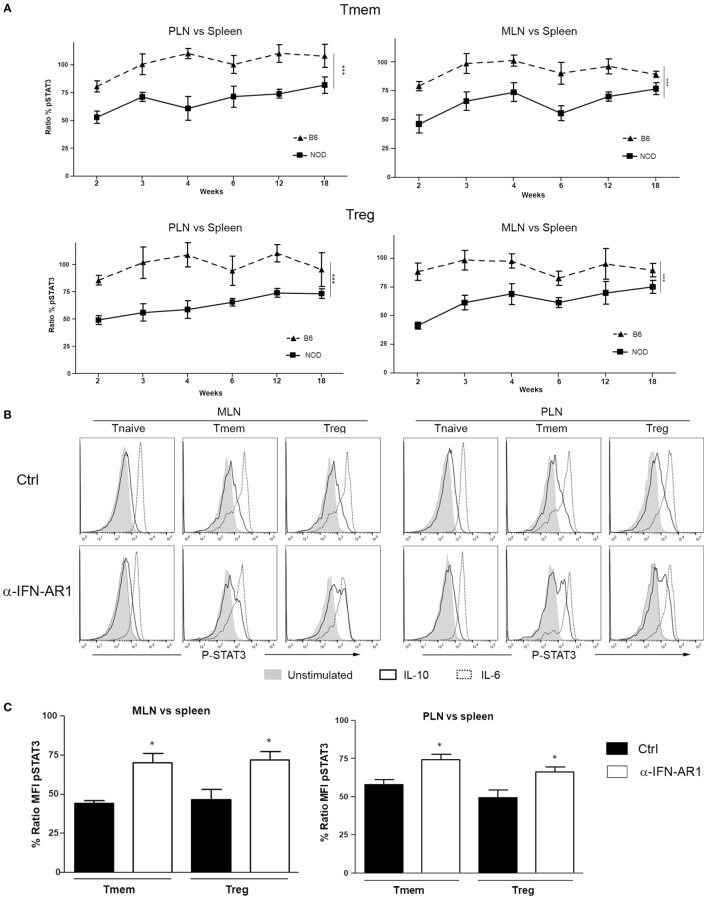
The localized inhibition of interleukin-10 (IL-10) signaling in T cells is sustained throughout the life of nonobese diabetic (NOD) mice and depends on IFN-β signaling. **(A)** P-STAT3 induction in response to IL-10 in T cell subsets of pancreatic lymph nodes (PLN), mesentheric lymph nodes (MLN), and spleen collected from NOD and C57BL/6 mice at the indicated age. Graph indicate the kinetic of P-STAT3 mean fluorescence intensity (MFI) ratio between the spleen and PLN or MLN (as indicated) memory T cells (Tmem) and regulatory T cell (Treg) after IL-10 stimulation. Final value was calculated by comparing the coefficient index of P-STAT3 between the two different organs, considering the levels in spleen as 100% of expression. Data of *n* = 4 mice per strain and per age point are shown and expressed as % of ratio MFI ± SEM. Differences between B6 and NOD were calculated using a two-way ANOVA (***p < 0.001). **(B,C)** Transient blockade of type-I interferons (TI-IFNs) signaling partially restores IL-10 signaling in T cells. NOD mice were treated with control or a blocking anti-IFNA-R mAb (0.5 mg on days 14 and 21 of age) and, on day 25, PLN, MLN, and spleen were extracted. Cells were left untreated or stimulated with IL-10 (40 ng/ml) or IL-6 (40 ng/ml) and the levels of P-STAT3 in CD4 T cell subpopulations measured *via* Phospho-flow. Representative histograms are presented in **(B)**. Cumulative results are shown in **(C)**, where graph bars represent the normalized P-STAT3 MFI ratio in Tmem or Treg after IL-10 stimulation between the spleen and PLN or MLN in control vs treated NOD mice. Ratio MFI was calculated by comparing the coefficient index of P-STAT3 between the two different organs, considering the levels in spleen as 100% of expression. Data of *n* = 4 mice per treatment group are shown and expressed as % of ratio MFI ± SEM, *p < 0.05, Mann–Whitney *U* test.

Knowing whether the inhibition of IL-10 signaling in T cells of PLN and MLN of NOD mice is sustained or altered over time is important for defining possible windows of therapeutic intervention. The accumulation of TI-IFN in PLN (13) appears maintained over time, though to a progressively lower level. As the inhibition of IL-10 signaling correlates with TI-IFN levels, we executed a longitudinal analysis of the response to IL-10 and IL-6 by Treg and Tmem cells of female NOD mice of different ages (weeks: 2, 3, 4, 6, 12, and 18). An identical analysis was performed in age-matched female wt B6 mice. Throughout the observation period, the ratio of IL-10 induced P-STAT3 in T cells between PLN/spleen and MLN/spleen (as shown in Figure 1) was significantly lower than that exhibited by B6 mice (Figure 7A). Interestingly, in 2-week-old B6 mice, the response to IL-10 of Tmem and Tregs in PLN and MLN was slightly lower than that observed in the spleen (<100%), but it quickly recovered and stabilized (Figure 7A).

### The Early Blockade of TI-IFN Signaling Partially Restores the Response to IL-10 in PLN and MLN Tmem and Tregs in NOD Mice

The reported delay and lower incidence of diabetes development in NOD mice treated at 2–3 weeks of age with an anti-IFN-AR1 mAb (13), together with the reversibility of IL-10 signaling inhibition we observed *in vitro* in T cells after removing IFN-β, are encouraging indications that the unbalanced immune regulation of NOD mice can be prevented or restored. To test if the therapeutic blockade of TI-IFN and alteration of IL-10 signaling were correlated *in vivo* in NOD mice, anti-IFN-AR1 mAb was administered on days 14 and 21 of age in female NOD mice and, on day 25, we measured the induction of P-STAT3 in PLN and MLN T cell subsets in response to *ex vivo* IL-10 treatment. Both Tmem and Treg populations in PLN and MLN of anti-IFN-AR-treated animals showed a significant recovery in P-STAT3 induction in response to IL-10 (Figures 7B,C). These results suggest that the therapeutic effect of early administration of anti-IFN-AR1 mAb in NOD mice may be due to the restoration of IL-10 signaling.

## Discussion

In addition to genetic susceptibility, type 1 diabetes development is clearly linked to still poorly defined environmental factors contributing to the initiation and/or progression of the disease. There is a growing number of reports that associate high levels of TI-IFN with the onset and progression of this pathology (8–10, 12–14). However, mechanistic insights on how TI-IFN would favor type 1 diabetes development are lacking, contributing to a degree of confusion in understanding this connection. In particular, how the accumulation of TI-IFN could result in a localized effect that enables the activity of diabetogenic T cells remains an enigma. The finding that dendritic cells of NOD mice produce higher levels of TI-IFN in response to TLR stimulation than cells of B6 mice (32) provides an important clue to the causes of localized and chronic TI-IFN accumulation in PLN reported previously (13). Based on our results, we propose that this chronic accumulation of TI-IFN in NOD mice, which we report for the first time also extends to the MLN, re-programs the response of CD4 Treg and Tmem to IL-10 and potentially diminishes the homeostatic regulation of diabetogenic cells. This phenomenon likely contributes to the progressive attack to the islets and development of the disease. These data are supported by the recovery of IL-10 signaling we observed after blockade of TI-IFN–IFNR1 interactions, a treatment that results in a reduction of the disease incidence and progression in NOD mice (13).

Our results provide some important clues on the molecular mechanism behind the inhibition of IL-10 signaling. The normal level of phosphorylation of STAT3 we measured in response to IL-6 in T cells pre-exposed to IFN-β suggested that the impairment observed in IL-10 signaling is not caused by a generalized reduction in the cytoplasmic availability of STAT3. The lack of upregulation of SOCS1 and SOCS3 (at either the mRNA or protein level) and the unaltered (if not slightly increased) expression of IL-10R in T cells we observed post-IFN-β exposure both *in vitro* and *in vivo* do not support regulation of P-STAT3 induction at this level. Our results suggest then that an IL-10 signaling negative regulator acting between the receptor and STAT3 phosphorylation is either being upregulated or activated. For example, direct or indirect alterations in the binding and phosphorylation of Jak1 and Tyk2 would regulate STAT3 activation (33); the involvement of molecules that interact with STAT3, including other members of the STAT family, should also be explored as they could interfere with its binding to IL-10R and its phosphorylation (30). Interestingly, human monocytes and macrophages primed with IFNα/β showed an increase in IL-10R1 and an increase in IL-10 signaling (34). However, these experiments were performed with an acute exposure (5 h) to TI-IFN. A prolonged exposure of human macrophages to TI-IFN promoted a switch in the signaling of IL-10 from activation of STAT3 to STAT1 (35, 36); in our settings, however, STAT1 phosphorylation was not appreciable in any condition tested (not shown). Overall, these observations suggest that IFN-β can have different consequences depending on the timing and context of exposure as well as on the target cell population, probably contributing to the (sometimes discordant) range of outcomes attributed to TI-IFN (37). Our ongoing work is now focused on dissecting these regulatory mechanisms and identify previously unappreciated modulators of IL-10 signaling.

The protective effect of Tofacitinib and Ruxolitinib we observed in our experiments indicates that signaling through the Jak/STAT pathway is involved in this specific effect of TI-IFN—though we cannot exclude the participation of other signaling routes (38). A deeper understanding of the molecular mediators of this phenomenon would be of crucial importance as some of these molecules could be targeted to control the development of diabetes. In fact, Jak1/Jak2 inhibition *in vivo* is effective at preventing, and reverting, established insulitis in NOD mice (39, 40). Improving the efficacy and safety of this type of intervention would be a major advancement for type 1 diabetes patients.

Our study shows that Tmem and Treg with impaired IL-10 signaling are present not only in PLN but also in the regionally close MLN. Paralleling the results from Rahman and colleagues (32), this phenomenon is specific for NOD animals, as in B6 mice, the response to IL-10 was not affected. This strain-specific effect in lymph nodes draining the gut (41) suggests a link with the recently discovered role of the microbiota in autoimmune diabetes development (2, 4); it could indicate an aberrant heightened chronic response to specific bacterial (or viral) derivatives that is not properly regulated. The initial impairment in IL-10 signaling we observed in 2-week-old B6 pups (similar to, but not to the same extent as, that of NOD mice, Figure 7), could indicate an initial adaptation phase of the newly generated pool of T cells (exposed to TI-IFN in the thymus) (42) to variations in the intestinal flora during the breast-feeding phase. This would suggest that the genetic predisposition of NOD mice encompasses a defect in establishing the proper balance in the response to microbiota derivatives in the gut-draining lymph nodes that ultimately affects the regulation of diabetogenic T cells by IL-10.

We observed that the impact of TI-IFN is not specific to the NOD genetic background, suggesting that this novel mechanism of alteration of immunoregulation could also contribute to the development of other disorders with a TI-IFN signature (43). This is a significant finding as the reduction of IL-10 signaling in Treg and Tmem populations has very deleterious effects on regulating autoreactivity (23–25). In animal models of diabetes (44, 45) as well as diabetic patients (46), Tregs exhibit reduced regulatory efficacy while effector T cells are resistant to regulation (44). Altogether, these results point toward a loss of regulation of autoreactive T lymphocytes as a key process in diabetes development. A selective targeting of this phenomenon, by either correcting the aberrant production of TI-IFN or by preventing its modulation of IL-10 signaling, could significantly improve the efficacy of some approaches currently being explored for the treatment of type 1 diabetes (47). Targeting IL-10 signaling has already been considered in the treatment of this disease (16, 17). However, inconsistent effects have been reported (48–51). Based on our results, we suggest that, rather than augmenting the concentration of IL-10, a targeted restoration of the IL-10 impact on diabetogenic cells (*via* a timed and localized intervention) would achieve more successful therapeutic outcomes. To this end, understanding how IL-10 modulates T cell function is necessary to identify the best strategy to recover an appropriate level of regulation but, to date, very little is known. We report here for the first time several genes (*Sphk1, LIGHT, Tarm1*, and *2B4*) that IL-10 induces in T cells. We used their expression as readout of IL-10 function, demonstrating the impact of pre-exposure to TI-IFN both *in vitro* and *in vivo* in NOD mice. Our future studies will be centered on understanding the transcriptional impact of IL-10 on T cells and the involvement of specific genes in the modulation of T cell functions. Moreover, the differential expression profile of the four genes between Tmem and Treg populations suggests a distinct role in each population; a property that could also reveal strategies to selectively impact these two subsets.

In summary, our study unveils the existence of a new molecular mechanism through which TI-IFN can alter T cell regulation and improves our understanding of IL-10-mediated control of Treg and Tmem cells. A deeper understanding of this phenomenon will very likely reveal novel points of intervention to restore the necessary immune regulatory network to potentiate the efficacy of immunotherapies for type 1 diabetes and possibly other autoimmune diseases.

## Ethics Statement

All animal experiments were conducted in accordance with the National Institutes of Health guide for use and care of laboratory animals, and under a protocol approved by the JHU Animal Care and Use Committee.

## Author Contributions

MI and GR developed the project, researched data, designed experiments, and wrote the manuscript. AA, MC, and BL contributed to experimental design and researched data. CT and VI researched data. WL and GB contributed to data interpretation, troubleshooting, and provided essential manuscript feedback. GR is the guarantor of this work and, as such, had full access to all the data in the study and takes responsibility for the integrity of the data and the accuracy of the data analysis.

## Conflict of Interest Statement

The authors declare that the research was conducted in the absence of any commercial or financial relationships that could be construed as a potential conflict of interest.

## Abbreviations

ALN, axillary lymph nodes; IL-10, interleukin-10; MFI, mean fluorescence intensity; MLN, mesentheric lymph nodes; NOD, nonobese diabetic; PLN, pancreatic lymph nodes; TI-IFN, type-I interferons; Tmem, memory T cells; Tregs, regulatory T cells.
