# Supplementary material for: Type-I Interferons Inhibit Interleukin-10 Signaling and Favor Type 1 Diabetes Development in Nonobese Diabetic Mice
Source: Front Immunol. 2018 Jul 16;9:1565. doi: 10.3389/fimmu.2018.01565 (PMC6054963; doi:10.3389/fimmu.2018.01565)
Supplement: Supplementary file 1 [file presentation_1.PDF]

Supplementary Material

Type-I interferons inhibit interleukin-10 signaling and favor type 1 diabetes development in NOD mice

Marcos Iglesias, Anirudh Arun, Maria Chicco, Brandon Lam, Conover Talbot, Vera Ivanova, W.P. A Lee, Gerald Brandacher, and Giorgio Raimondi*

*** Correspondence:** Giorgio Raimondi: g.raimondi@jhmi.edu

Supplementary Figures

- **Supplementary Figure 1.** Differential gene expression of IFN-α and IFN-β in tissues of C57BL/6 and NOD mice.
- **Supplementary Figure 2**. Dose-effect of IFN-α and IFN-β inhibiting IL-10 signaling in T cells.
- **Supplementary Figure 3**. Genes tested for expression/modulation after IL-10 stimulation in T cells.
- **Supplementary Figure 4**. Effect of Tofacitinib and Ruxolitinib in cytokine signaling.
- **Supplementary Figure 5**. Response of IFN-AR1$^{-/-}$ T cells to IL-10 stimulation

Supplementary Tables

- **Table supplementary 1**. Sequence of primers used in RT-PCT studies

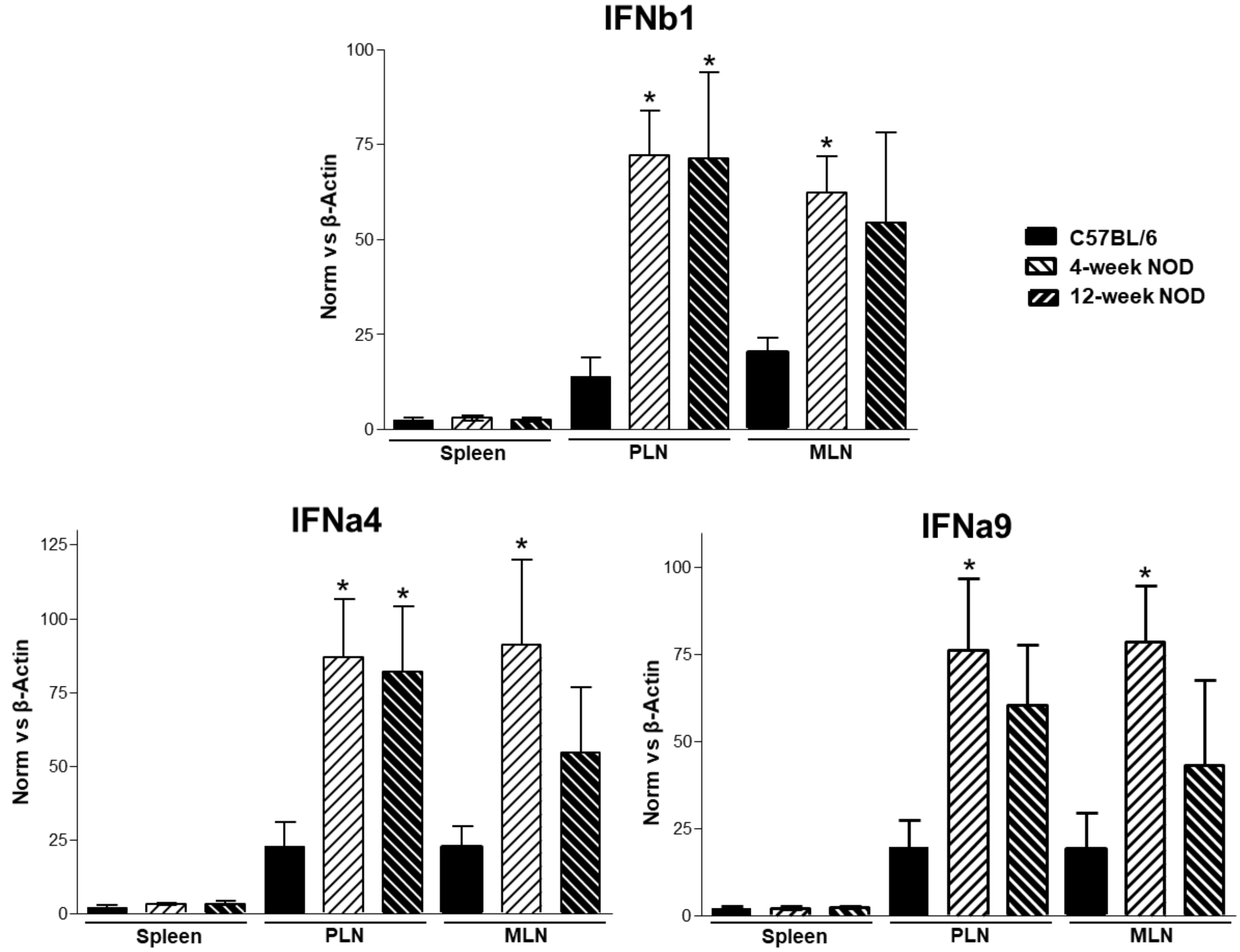

Supplementary Figure 1. Differential gene expression of IFN-α and IFN-β in tissues of C57BL/6 and NOD mice. Spleen, PLN, and MLN of 4 week old C57BL/6 and 4 or 12 week old NOD mice were separately harvested. Tissues were then lysed and mRNA levels of IFNa4, IFNa9 and IFNb1 genes were measured by qPCR. ΔΔCt method was used to calculate their relative expression and normalized to β-Actin. The graph bars show the fold change among the indicated tissues and mouse strains ±SEM where *$p<0.05$ in Mann-Whitney U test was considered with statistically significance. Data shows the average of n=4 animals per group.

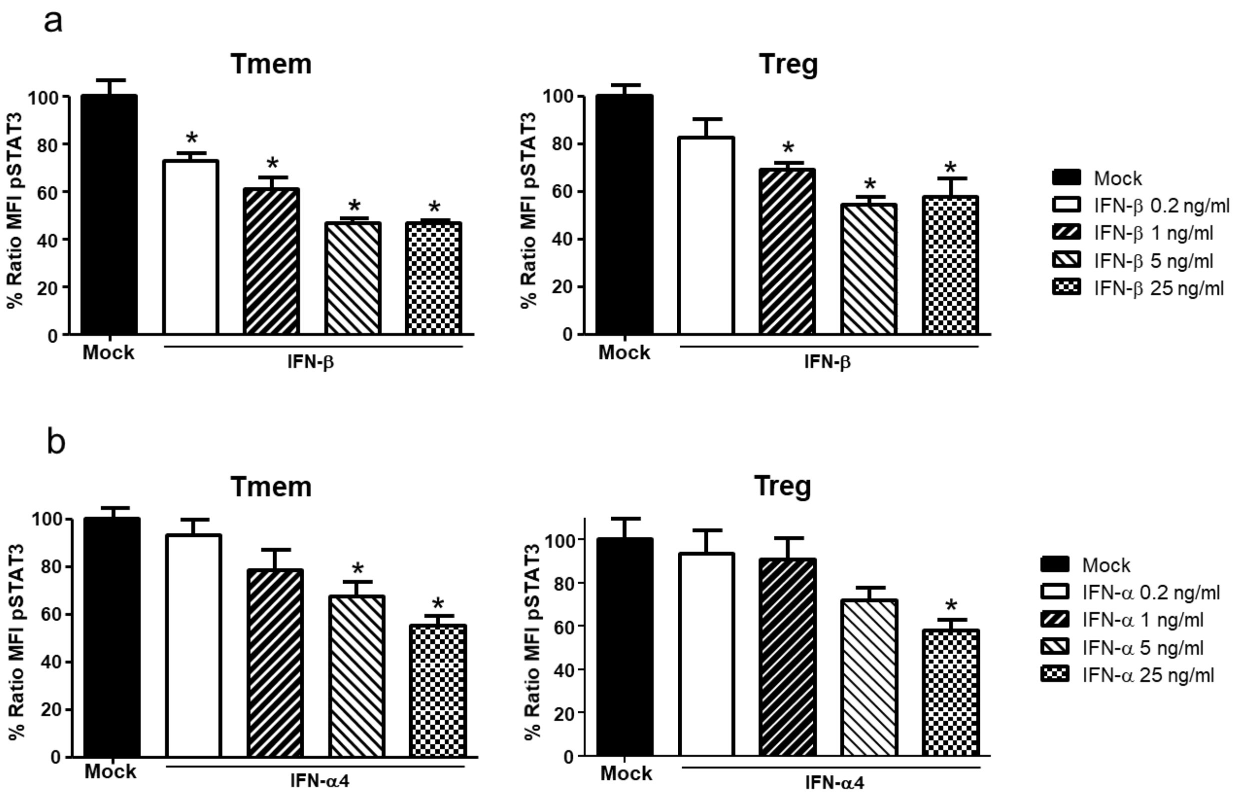

Supplementary Figure 2. Dose-effect of IFN-α and IFN-β inhibiting IL-10 signaling in T cells. Purified T cells from C57BL/6 mice were cultured for 48h in RPMI complete media in the absence (mock culture) or presence of different concentrations of IFN-β (**a**) or IFN-α4 (**b**) (0.2-25ng/ml), and then rested in cytokine-free media for six additional hours. Cells were then left untreated or stimulated with IL-10 (40 ng/ml) for 20’. After a fixation step, the levels of P-STAT3 in CD4 T cell subpopulations (Tmem: $CD4^{+}CD44^{hi}Foxp3^{-}$, Treg: $CD4^{+}Foxp3^{+}$) were measured by phospho-flow. The graph bars compare the percentage of P-STAT3 MFI ratio in Tmem and Treg cells between the groups exposed to IFN-β/α and mock condition (considered as 100% of response) after IL-10 stimulation. Ratio MFI was calculated comparing the coefficient index of P-STAT3 after stimulation between each of the IFN-β/α exposed groups and mock. Data of n=4 individual experiments are shown and expressed as % of Ratio MFI±SEM, *$p<0.05$, Mann-Whitney U test.

a

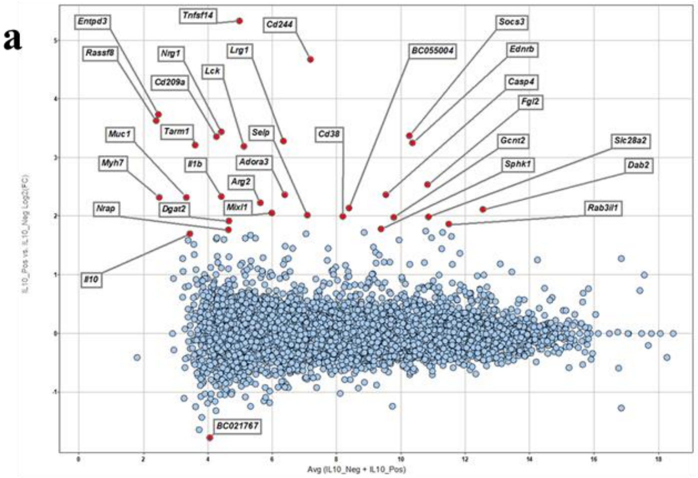

b

Gene name	Characteristic and function in T cells of the protein encoded (if known)
**Lck**	Tyrosine kinase associated to CD4 and CD8 that assists in signaling from the T cell receptor complex
**CD244 (2B4)**	Member of SLAM receptors with inhibitory function
**Tnfsf14 (LIGHT)**	Surface molecule, co-regulatory factor in the activation of T cells
**CD38**	T cell receptor and ectoenzyme. Activatory molecule
**Casp4**	Intracellular molecule with a role in cell apoptosis
**SOCS3**	Member of Suppressor of Cytokine signaling (SOCS) family. Negative regulators of cytokine signaling
**TARM1**	Costimulatory ITAM receptor identified in monocytes and neutrophils. Role in inflammation and their interaction with CD4 T cells..
**Sphk1**	Role in T cell proliferation and migration
**SELP**	Selectine-P. Present in platelets and endothelial cells. Role in their interaction with lymphocytes after activation
**Fgl2**	Produced by both CD4+ and CD8+ T cells. Modulatory function

Supplementary Figure 3. Genes tested for expression/modulation after IL-10 stimulation in T cells. (**a**) Re-analysis of the RNAseq project evaluating mouse macrophage gene expression in response to IL-10 stimulation, GSE49449, deposited in the NCBI Gene Expression Omnibus (GEO) database. A two-tailed one-way ANOVA was performed to determine IL-10-treated versus untreated cells' differential gene expression. This differential expression was then evaluated for the standard deviation, σ or SD, of each gene's log2 fold change from the mean, of zero or unchanged. The 29 genes highly upregulated (>6SD) are indicated. (b) The table shows the 10 genes among the 29 indicated in (a), that we found associated to T lymphocytes. Characteristics and function (if known) of the protein encoded are indicated.

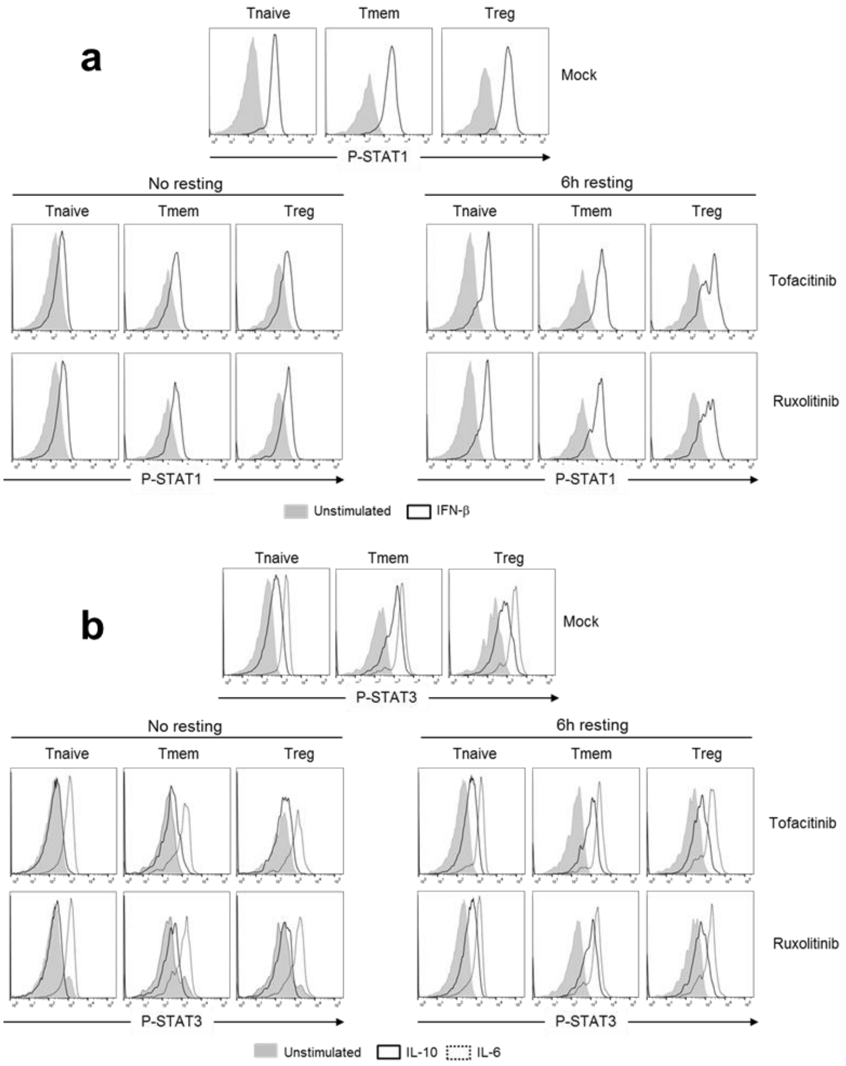

Supplementary Figure 4. Effect of Tofacitinib and Ruxolitinib in cytokine signaling. Purified T cells from C57BL/6 mice were cultured for 48h in RPMI complete media with or without Tofacitinib (25μM) or Ruxolitinib (5μM), and then directly stimulated or rested in cytokine-free media for additional 6 h. Cells in every of the mentioned conditions were either left untreated or stimulated with IFN-β for 30 min (**a**) or with IL-10 (40 ng/ml) or IL-6 (40 ng/ml) for 20 min (**b**). Representative histograms show the levels of P-STAT-1 (a) or P-STAT3 (b) after stimulation of the indicated CD4 T cell subpopulations (Tnaive: $CD4^{+}CD44^{low}Foxp3^{-}$, Tmem: $CD4^{+}CD44^{hi}Foxp3^{-}$ and Treg: $CD4^{+}Foxp3^{+}$). Results are representative of n=3 independent experiments.

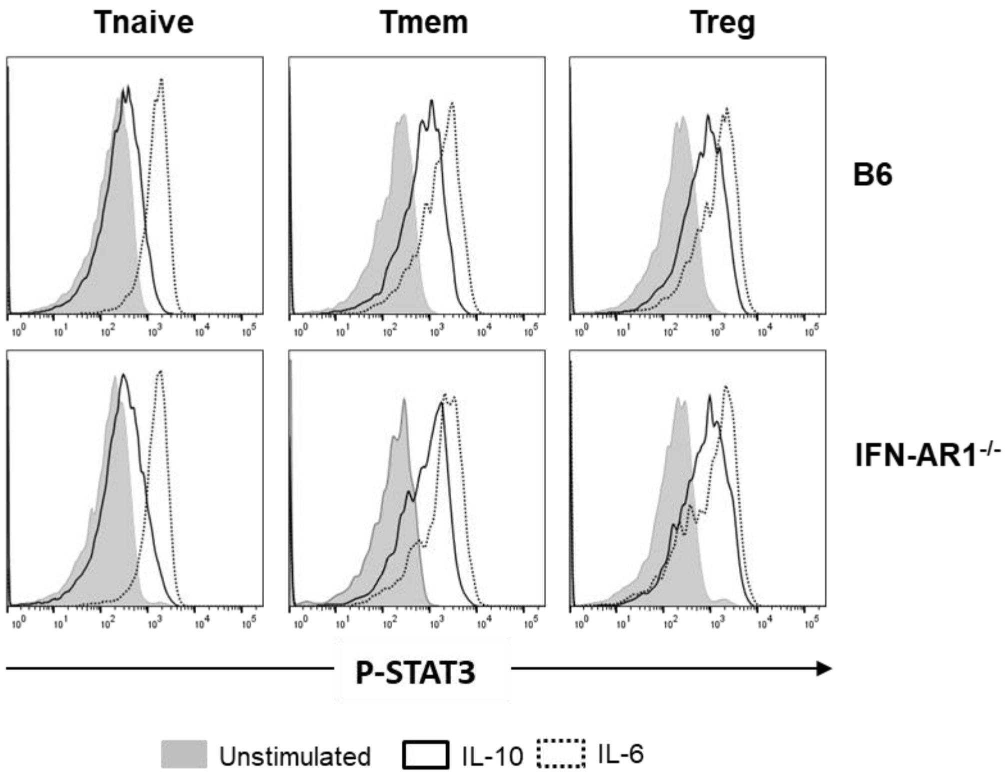

Supplementary Figure 5. Normal response of IFN-AR1$^{-/-}$ T cells to IL-10 stimulation. Freshly purified T cells from C57BL/6 and IFN-AR1$^{-/-}$ mice were either left untreated or stimulated with IL10 (40 ng/ml) or IL-6 (40 ng/ml) for 20'. The levels of P-STAT3 after stimulation in the indicated CD4 T cell subpopulations were measured by phospho-flow. Representative histograms show P-STAT3 levels in Tnaive: CD4^{+}CD44^{low}Foxp3^{-}, Tmem: CD4^{+}CD44^{hi}Foxp3^{-} and Treg: CD4^{+}Foxp3^{+} cells after stimulation. Results are representative of n=3 independent experiments.

Supplementary Tables

Table Supplementary 1. Sequence of primers used in RT-PCT studies

Gene Target	Direction	Sequence (5' → 3')	Source
Tnfsf14 (LIGHT)	Forward	TCTCTAGGAAAGCCCAGATCG	Harvard Primer Bank ID: 9507195a1
	Reverse	GTCCACACCAGGAGTGAGC	
Sphk1	Forward	AAAATACTGAGAAACTCGGTCGG	Harvard Primer Bank ID: 3659692a1
	Reverse	GCATCGCTTCTTAAAGTCCAGA	
CD244 (2B4)	Forward	CTCGGGGCCATCATTTGTTTC	Harvard Primer Bank ID: 3551962a1
	Reverse	GCTAGAAGGGAGCTGAACATCA	
Tarm1	Forward	TCTAGGCTCCTTTCCCTTCTC	Harvard Primer Bank ID: 28893513a1
	Reverse	GGGGTGGGGCTCTTACATT	
SOCS1	Forward	GACACTCACTTCCGCACCTT	Ref below (1)
	Reverse	GAAGCAGTTCCGTTGGCGACT	
SOCS3	Forward	ATGGTCACCCACAGCAAGTTT	Harvard Primer Bank ID: 6671758a1
	Reverse	TCCAGTAGAATCCGCTCTCCT	
IFNa4	Forward	TGATGAGCTACTACTGGTCAGC	Harvard Primer Bank ID: 6754294a1
	Reverse	GATCTCTTAGCACAAGGATGGC	
IFNa9	Forward	ATGGCTAGGCCCTTTGCTTTC	Harvard Primer Bank ID: 6754298a1
	Reverse	CAGTTCCTTCATCCCGACCAG	
IFNb1	Forward	CAGCTCCAAGAAAGGACGAAC	Harvard Primer Bank ID: 6754304a1
	Reverse	GGCAGTGTAACTCTTCTGCAT	
B-Actin	Forward	GGCTGTATTCCCCTCCATCG	Harvard Primer Bank ID: 6671509a1
	Reverse	CCAGTTGGTAACAATGCCATGT	
GAPDH	Forward	GGGAAGCCCATCACCATCT	Ref below (2)
	Reverse	CGACATACTCAGCACCGGC	

Appropriate primer sequence was obtained from http://pga.mgh.harvard.edu/primerbank/ except for:
(1) Collins, EL et al. J Immunol. 2011 Sep 1; 187(5): 2666–2676.
(2) Revert, F et al. Am J Pathol. 2007 Nov; 171(5): 1419–1430.

All genes were amplified using the same amplification program that consisted of: one cycle at 95°C for 15 min followed by forty five cycles at 94°C for 15 s, 60°C for 30 s and72^{o}C for 30 s; and concluding with a dissociation stage. The threshold for detection was set within the linear phase of the logarithmic amplification plot.
